# Physical Fitness Index for Older Adults in a Chinese Retirement Community

**DOI:** 10.3390/sports14090369

**Published:** 2026-09-01

**Authors:** Jiahao Li, Zhifan Ye, Jiali Lu, Huizhi Yang, Huiping Yan, Yifan Lu

**Affiliations:** 1 School of Sports Medicine and Rehabilitation, Beijing Sport University, Beijing 100084, China; leejhfighting@163.com (J.L.); yzf_1204@163.com (Z.Y.);; 2 Key Laboratory of Sports and Physical Fitness of the Ministry of Education, Beijing Sport University, Beijing 100084, China

**Keywords:** older adults, physical fitness, assessment, fitness index

## Abstract

Background: Physical fitness is important for health and independence in older adults, but existing systems do not always provide a continuous multidomain score. Objective: To develop and preliminarily evaluate a Physical Fitness Index (PFI) for older adults in a Chinese retirement community. Methods: A total of 561 participants aged 65–89 years completed assessments of body composition, strength, endurance, balance, mobility, and flexibility. Scores were normalized within sex and 5-year age groups and combined using a fixed radar-axis order. Results: The PFI averaged 39.24 ± 13.43 points (range, 4.16–84.47). In a reduced surrogate regression model, R^2^ was 0.825 and ten-fold cross-validation yielded an RMSE of 5.79 points and an MAE of 4.33 points. Conclusion: The PFI provides a continuous overall score with a domain profile, but its calibration and measurement properties require external and longitudinal validation.

## 1. Introduction

Functional fitness—including strength, flexibility, balance, coordination, and aerobic capacity—is essential for independence and health in older adults [1]. Its assessment is increasingly important as populations age. By the end of 2025, China had 323.38 million residents aged ≥60 years (23.0% of the population), including 223.65 million aged ≥65 years (15.9%) [2]. Worldwide, the number of adults aged ≥80 years is projected to exceed the number of infants by the mid-2030s [3]. Assessment systems that stop at age 79 may therefore offer limited guidance for the oldest-old.

The consequences of declining physical fitness are not merely statistical. Reduced strength, endurance, and balance are core features of frailty and are associated with disability, falls, and loss of independent living [4,5,6]. Habitual physical activity and social factors, such as living arrangement, further shape which forms of exercise older adults sustain and how satisfied they are with their health status [7,8,9]. Because global guidance now frames physical activity promotion as a public-health priority across the life course [10], assessment tools capable of resolving fitness at an individual level—rather than only classifying people into broad risk categories—have practical value for both clinical monitoring and policy-level surveillance.

Several instruments already capture different facets of fitness in older adults. The American Alliance for Health, Physical Education, Recreation and Dance (AAHPERD) battery and the Senior Fitness Test (SFT) assess multiple domains, and the China National Physical Fitness Evaluation Standards (CNPFES) provide age- and sex-specific scores [11,12,13]. Yet the SFT does not yield a single continuous index, and the CNPFES norms mainly cover ages 60–79. Extending these age ranges would require new reference data, but this alone would not produce a continuous score paired with a multidomain profile.

Other composite approaches, such as the Short Physical Performance Battery, SFT-based Z-scores, independence-related cut-offs, and the WHO intrinsic-capacity framework, have also been proposed [1,12,13,14,15,16]. These tools are useful for risk stratification or geriatric evaluation, but none combines eight physical-fitness domains into a single continuous score with a visual profile. The PFI was developed to occupy this complementary niche rather than to replace existing systems.

Accordingly, this study developed and preliminarily evaluated a continuous, multidomain PFI in relatively healthy adults aged 65–89 years from a Chinese retirement community.

## 2. Methods

### 2.1. Study Design and Participants

This study involved a retrospective analysis of prospectively collected cross-sectional data. Participants were recruited from a retirement community in Langfang, Hebei Province, China, between 1 September and 30 September 2021.

Participants were recruited through on-site health lectures and registered voluntarily; thus, recruitment was non-random and convenience-based.

Of 797 registrants, 94 were excluded for medical or safety reasons and 142 for incomplete assessments or data required to construct the PFI, leaving 561 participants.

Recruitment was open to adults aged ≥60 years, but the final sample contained no participants aged 60–64 or ≥90 years; the observed range was 65–89 years.

Participants were eligible if they were aged ≥60 years; were able to ambulate independently without an assistive device; were able to understand and follow standardized assessment instructions; were classified as having low or moderate cardiovascular risk following pretest screening; and provided written informed consent.

The exclusion criteria were the presence of a cardiac stent or pacemaker; an artificial joint or a fracture within the preceding 3 months; diabetes mellitus; osteoarthritis or another musculoskeletal condition that substantially affected movement; asthma; malignant tumors; chronic heart failure; severe depression or another psychiatric disorder; or any other medical or safety condition that prevented the safe completion of the assessment battery.

The criteria were intended to reduce testing risk and interference from conditions directly limiting movement. Hypertension and stable cardiac disease were permitted when screening identified no exercise contraindication. The sample therefore represented relatively healthy, independently ambulatory older adults rather than the wider older population.

The Physical Activity Readiness Questionnaire and a study-specific exclusion checklist were administered to determine eligibility and ensure participant safety [17]. All participants provided written informed consent before participation.

The study protocol was conducted in accordance with the Declaration of Helsinki and was approved by the Ethics Committee of Sport Science Experimentation at Beijing Sport University (Approval No. 2020082H; 1 July 2020). Data were collected in September 2021, and the study was retrospectively registered in the Chinese Clinical Trial Registry (ChiCTR2200064801) on 19 October 2022. All analytical data were anonymized before analysis.

### 2.2. Assessment Procedures and Measurements

#### 2.2.1. Pretest Screening and Safety Procedures

Before testing, participants completed the Physical Activity Readiness Questionnaire and exclusion checklist, performed a standardized warm-up, and underwent blood-pressure, heart-rate, and other safety checks.

All assessments were completed on the same day without a fixed order. Rest intervals were 5–10 min and were extended for fatigue or discomfort. Assessors explained and demonstrated each test, provided practice trials when required by the protocol, and monitored participants throughout. No adverse events occurred, and no participant used a walking aid.

#### 2.2.2. Assessor Training and Procedural Standardization

Fifteen graduate students with sports-medicine backgrounds administered the assessments. They had completed relevant coursework and 1 month of standardized training, including inter-assessor consistency exercises. Each test type was administered by the same designated assessor throughout data collection.

#### 2.2.3. Cognitive Assessment

Cognitive function was assessed with the 30-point Mini-Mental State Examination (MMSE), with higher scores indicating better global cognition [18]. Assessors also confirmed participants’ ability to understand instructions and complete testing safely.

MMSE was a baseline descriptive variable, not a PFI component. Data were available for 458 participants; missing values were not imputed.

#### 2.2.4. Physical Fitness Assessments

The assessment battery included body mass index (BMI), the 6-Minute Walk Test, 2-Minute Step Test, 30-Second Chair Stand Test, Arm Curl Test, Handgrip Strength Test, Chair Sit-and-Reach Test, Back Scratch Test, 8-Foot Up-and-Go Test, and Berg Balance Scale [19].

The measures were selected to cover prespecified domains relevant to independent functioning in older adults. Most procedures were drawn from the validated Senior Fitness Test battery or other established protocols; the 6-Minute Walk Test and Berg Balance Scale followed their respective methodological sources [19,20,21]. When two tests represented the same domain, the final indicator was selected using the prespecified criteria described in Section 2.3.2.

Body mass index: Body height and weight were measured using an RGZ-120 height-and-weight scale. Participants were instructed to avoid food and drink for at least 2 h before measurement and were measured wearing light clothing and without shoes. BMI was calculated as weight in kilograms divided by height in meters squared (kg/m^2^).

Six-Minute Walk Test: Aerobic endurance was assessed using the 6-Minute Walk Test. The test was conducted on a marked rectangular course measuring 20 yards in length and 5 yards in width, with one complete circuit corresponding to 50 yards. Participants were instructed to walk continuously for 6 min and to cover as much distance as safely possible without running. The assessor recorded the number of completed laps and any additional distance covered. Total walking distance was converted to meters. No walking aid was used. Because the course configuration differed from the straight corridor commonly recommended in the ATS protocol, the actual field procedure is reported explicitly and should not be interpreted as strict adherence to the ATS protocol [20].

The Two-Minute Step Test was performed with the target stepping height defined as the midpoint between the patella and iliac crest. Participants marched in place for 2 min, and the number of times the right knee reached the target was recorded.

The Thirty-Second Chair Stand Test was performed using a 47 cm-high chair. Participants rose to a full standing position and returned to sitting as many times as possible within 30 s, with correctly completed repetitions recorded.

Arm Curl Test: Women used a 5-lb dumbbell and men used an 8-lb dumbbell [22]. After one or two practice repetitions, participants completed as many correctly performed arm curls as possible within 30 s. The number of correctly completed repetitions was recorded.

Handgrip strength: Maximum isometric handgrip strength was measured using an electronic handgrip dynamometer. Participants stood naturally with their feet approximately shoulder-width apart. The tested arm remained alongside the body with the elbow fully extended, and body swinging or compensatory movements were not permitted. Participants squeezed the dynamometer as forcefully as possible and maintained the maximal effort for approximately 3–5 s. Each hand was tested twice, with an interval of at least 30 s between repeated measurements. The highest value obtained from either hand was used in the analysis. All handgrip assessments were administered by the same designated assessor.

Chair Sit-and-Reach Test: Participants sat near the front edge of a 43 cm high chair, extended one leg with the heel on the floor, and reached both hands toward the toes. After practice trials with both legs, the preferred leg was selected. Two recorded trials were conducted. Negative values indicated that the fingertips did not reach the toes, whereas positive values indicated that the fingertips extended beyond the toes. The better trial was used in the analysis.

Back Scratch Test: Participants reached one hand over the shoulder and the other hand upward behind the back and attempted to bring the middle fingers together. After two practice trials, two recorded trials were conducted. Negative values represented a gap between the fingers, whereas positive values represented an overlap. The better trial was used in the analysis.

Eight-Foot Up-and-Go Test: Participants began seated in a chair and, following the start command, stood up, walked 8 ft around a marker, returned to the chair, and sat down. Completion time was recorded in seconds. No assistive device was used. Because a shorter completion time represented better performance, reverse normalization was applied during score construction. The test was evaluated as a candidate indicator during the indicator-screening process.

Berg Balance Scale: Functional balance was assessed using the standardized 14-item Berg Balance Scale [21]. Each item was scored from 0 to 4, producing a total score of 0–56, with higher scores indicating better balance.

### 2.3. Statistical Analysis

The statistical analysis followed four sequential stages: description of the study sample, selection of representative indicators within each physical fitness domain, construction and preliminary evaluation of the PFI, and descriptive comparison of the PFI with existing physical fitness assessment systems. The study was regarded as an initial development and preliminary evaluation of the PFI rather than a comprehensive psychometric validation study.

#### 2.3.1. Descriptive Statistics

Continuous variables were summarized as mean ± standard deviation. Distributional characteristics were examined using the Shapiro–Wilk test and graphical inspection. Sex differences were examined using an independent-samples *t*-test, Welch’s *t*-test, or Wilcoxon rank-sum test, as appropriate. Potential age outliers were assessed using Tukey fences (Q1 − 1.5 × IQR and Q3 + 1.5 × IQR). Statistical significance was set at *p* < 0.05.

For MMSE analysis, only scores within the theoretical range of 0–30 points were treated as valid. No MMSE score outside this range was identified in the final analytical sample. Missing values were not imputed. MMSE scores were summarized as mean ± standard deviation, and the difference between women and men was examined using Welch’s independent-samples *t*-test.

#### 2.3.2. Selection of Representative Physical Fitness Indicators

Candidate measures covered eight domains. When two tests represented one domain, selection considered data completeness, safety and feasibility, ease of standardized administration, dispersion (CV and IQR), and overlap between tests. CV and IQR were supporting descriptive statistics, not measures of validity.

Within-domain associations between candidate measures were assessed using Spearman rank correlations. Correlations among the retained indicators were displayed using chart. Correlation in the PerformanceAnalytics R package. All analyses were performed using R software (version 4.4.2).

#### 2.3.3. Normalization and Calculation of the Physical Fitness Index

To account for age- and sex-related differences in physical fitness, participants were stratified into 5-year age intervals, and normalization was conducted separately by sex within each age group. For indicators in which a higher raw value represented better performance, normalization was performed using y = (x − xmin)/(xmax − xmin), where xmin and xmax were the observed minimum and maximum values within the corresponding sex-by-age subgroup.

Sample-based min-max normalization was used because no single external reference system provided compatible sex- and age-specific norms for all eight indicators, particularly for adults aged 80 years or older. Existing systems also differed in populations, protocols, units, and scoring directions; combining them would have produced inconsistent scaling across domains. The resulting PFI was therefore intended for internal comparison within the development sample, not as a population norm or clinical threshold.

For time-based indicators in which a lower raw value represented better performance, such as the 8-Foot Up-and-Go Test, reverse normalization was applied using y = (xmax − x)/(xmax − xmin). BMI was scored separately because both excessively low and excessively high values may represent less favorable body composition. The highest score was assigned to the prespecified appropriate BMI interval, with scores decreasing toward both lower and higher extremes.

After normalization, the axes were fixed clockwise as follows: 6-Minute Walk Test, handgrip strength, Back Scratch Test, Chair Sit-and-Reach Test, 8-Foot Up-and-Go Test, 30-Second Chair Stand Test, Berg Balance Scale, and BMI. This order grouped conceptually related domains adjacently while preserving a single prespecified arrangement for every participant. Following the radar (polygon-area) approach to presenting multivariate profiles [23], the PFI was defined as the radar-polygon area relative to the theoretical maximum area, multiplied by 100; its theoretical range was 0–100. Because polygon area depends on adjacent-axis pairings, the fixed order is part of the PFI definition and must be retained for reproduction.

#### 2.3.4. Preliminary Evaluation of the Physical Fitness Index

The preliminary evaluation focused on the distributional characteristics, internal sensitivity, and structural behavior of the PFI within the development sample. Descriptive statistics included the mean, standard deviation, median, interquartile range, minimum, maximum, and CV. Floor and ceiling effects were evaluated as the proportions of participants scoring in the lowest and highest 10% of the theoretical score range; a proportion exceeding 15% was considered indicative of a potential floor or ceiling effect.

Associations between the PFI and its components were examined using Pearson or Spearman coefficients, as appropriate. Component-deletion sensitivity analyses were conducted. Axis-order sensitivity was assessed using 1000 random permutations; each alternative score was compared with the fixed-order PFI using the mean absolute difference and Pearson and Spearman correlations.

#### 2.3.5. Comparison with Existing Physical Fitness Assessment Systems

The PFI was descriptively compared with the China National Physical Fitness Evaluation Standards and the Senior Fitness Test. Comparisons were restricted to participants for whom corresponding reference scores could be derived. Where necessary, the reference age group most closely corresponding to the study population was used to facilitate comparison across systems.

Score distributions were visualized using density plots generated with the ggdensity function in the ggpubr R package. Distributional characteristics were summarized using the mean, standard deviation, median, IQR, CV, skewness, kurtosis, and floor and ceiling effects. These analyses were intended to provide a descriptive comparison of score distributions, variability, and potential discriminatory capacity rather than to establish the superiority or external validity of one assessment method over another.

#### 2.3.6. Application Model for Estimating the Physical Fitness Index

A multiple linear regression model was developed as a simplified surrogate for the geometric PFI. Candidate predictors were the raw values of BMI, 30-Second Chair Stand, handgrip strength, 6-Minute Walk, Berg Balance Scale, Chair Sit-and-Reach, and Back Scratch, together with age and sex. The raw 8-Foot Up-and-Go time was reviewed separately and excluded from the final surrogate equation because its coefficient was directionally inconsistent, non-significant, and did not materially improve model fit. The 8-Foot Up-and-Go remained a reverse-scored component of the geometric PFI.

Multicollinearity among predictors was assessed using variance inflation factors, and ten-fold cross-validation was used to assess the internal stability and predictive performance of the regression model. The regression equation was regarded as a simplified surrogate for estimating the already constructed geometric PFI. Cross-validation assessed the internal predictive performance of the surrogate equation and was not interpreted as external validation of the PFI.

## 3. Results

### 3.1. Subject Information

A total of 561 participants (215 men and 346 women) were included, with a mean age of 80.52 ± 5.71 years (median, 81; IQR, 77–85; range, 65–89). The age groups comprised 28 participants aged 65–69 years (5.0%), 66 aged 70–74 (11.8%), 99 aged 75–79 (17.6%), 206 aged 80–84 (36.7%), and 162 aged 85–89 (28.9%). Thus, 193 participants (34.4%) were aged 65–79 years. The Tukey age limits were 65–97 years, so no participant was classified as an age outlier. All participants met the prespecified eligibility criteria and were retained. MMSE data were available for 458 participants (81.6%), with no sex difference (*p* = 0.834; Table 1).

Data are presented as mean ± SD. Sex differences were examined using an independent-samples *t*-test, Welch’s *t*-test, or Wilcoxon rank-sum test, as appropriate. MMSE data were available for 458 participants.

### 3.2. Indicator Selection and PFI Construction

#### 3.2.1. Screening for Physical Fitness

The indicators tested were categorized into 8 categories: body composition, lower body strength, upper body strength, endurance, static balance, dynamic balance, lower body flexibility and upper body flexibility. As shown in Table 2, two indicators were included in both the upper body strength and endurance capacity.

**Figure 1:**
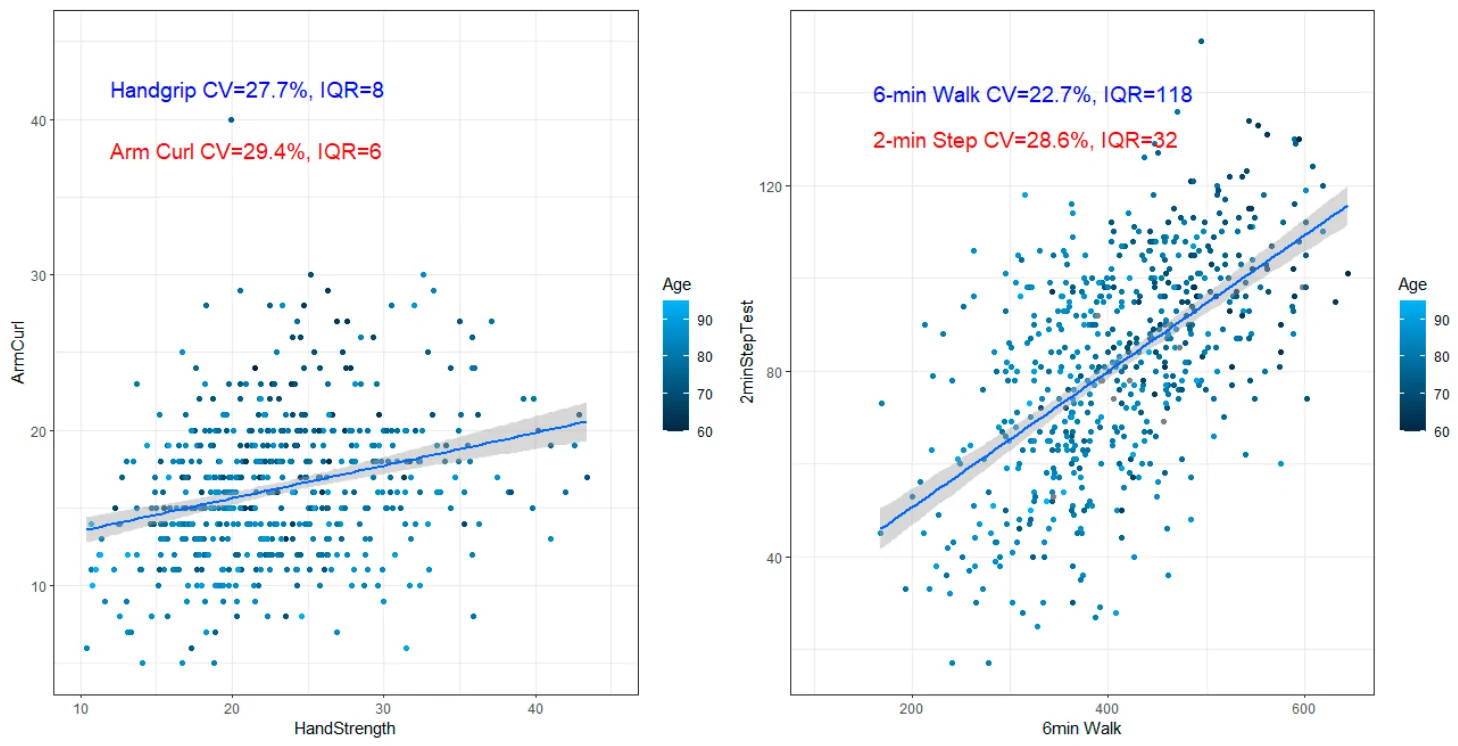
(**right**) Distribution characteristics of 6 min walk test and 2 min step test. (**left**) Distribution characteristics of handgrip strength and arm curl.

For domains represented by two tests, selection considered data completeness, safety and feasibility, ease of administration, score dispersion, and within-domain correlations (Table 3 and Figure 1).

For aerobic endurance, the 6-Minute Walk Test had complete data (n = 561), was self-paced and simple to administer, and showed adequate dispersion (CV = 22.69%; IQR = 118.0 m). The 2-Minute Step Test had 469 valid observations, and the tests were moderately correlated (Spearman ρ = 0.554, *p* < 0.001). The 6-Minute Walk Test was retained based on completeness, safety-related feasibility, administration, and dispersion.

For upper-body strength, handgrip strength had complete data (n = 561), low mobility demands, standardized administration, and adequate dispersion (CV = 27.74%; IQR = 8.0 kg). The Arm Curl Test had 554 valid observations, and the tests were modestly correlated (Spearman ρ = 0.278, *p* < 0.001). Handgrip strength was therefore retained.

Accordingly, the indicators retained for construction of the PFI were BMI for body composition, the 30-Second Chair Stand Test for lower-body strength, handgrip strength for upper-body strength, the 6-Minute Walk Test for aerobic endurance, the Berg Balance Scale for static balance, the 8-Foot Up-and-Go Test for dynamic mobility, the Chair Sit-and-Reach Test for lower-body flexibility, and the Back Scratch Test for upper-body flexibility.

#### 3.2.2. Construction of the Physical Fitness Index

The PFI used the fixed clockwise order specified in Methods. The mean fixed-order PFI was 39.24 ± 13.43 points (range, 4.16–84.47). Across 1000 random axis permutations, the mean absolute difference from the fixed-order score averaged 1.88 points; the mean Pearson correlation was 0.984 and mean Spearman correlation was 0.984. Axis order affected absolute values but rankings were generally stable, supporting the need to retain the prespecified order.

Sex- and age-specific normalization used the observed minimum and maximum values; thus, the PFI is a relative score calibrated to the development sample rather than a population norm.

**Figure 2:**
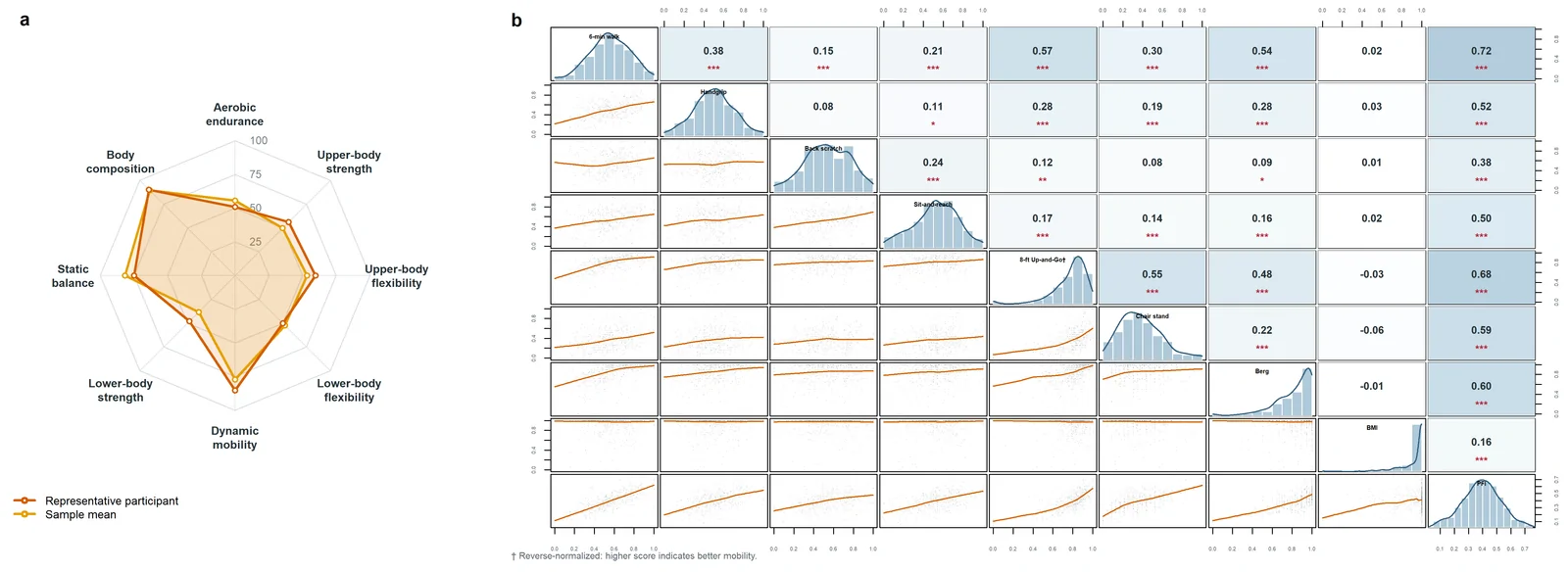
(**a**) Corrected radar profile using the fixed axis order. Red indicates a representative participant, and orange indicates the sample mean. (**b**) Spearman correlation matrix of the eight directionally harmonized component scores and the PFI. Higher component scores indicate better performance; the 8-Foot Up-and-Go Test was reverse-normalized. * *p* < 0.05, ** *p* < 0.01, *** *p* < 0.001.

All component indicators were associated with the PFI (Figure 2b). Because each indicator contributed to the score, these associations describe internal score behavior rather than construct validity. BMI had the weakest association but was retained to represent body composition.

Removing BMI changed the area-based score (*p* < 0.05); this exploratory sensitivity result was not interpreted as evidence of validity.

After reverse normalization, the 8-Foot Up-and-Go score was positively correlated with the Berg Balance Scale score (Spearman ρ = 0.48, *p* < 0.001) and with the PFI (Spearman ρ = 0.68, *p* < 0.001). Both measures were retained because they represented dynamic mobility and static balance, respectively; component-deletion results were treated as exploratory.

### 3.3. Comparison of the Physical Fitness Index

**Figure 3:**
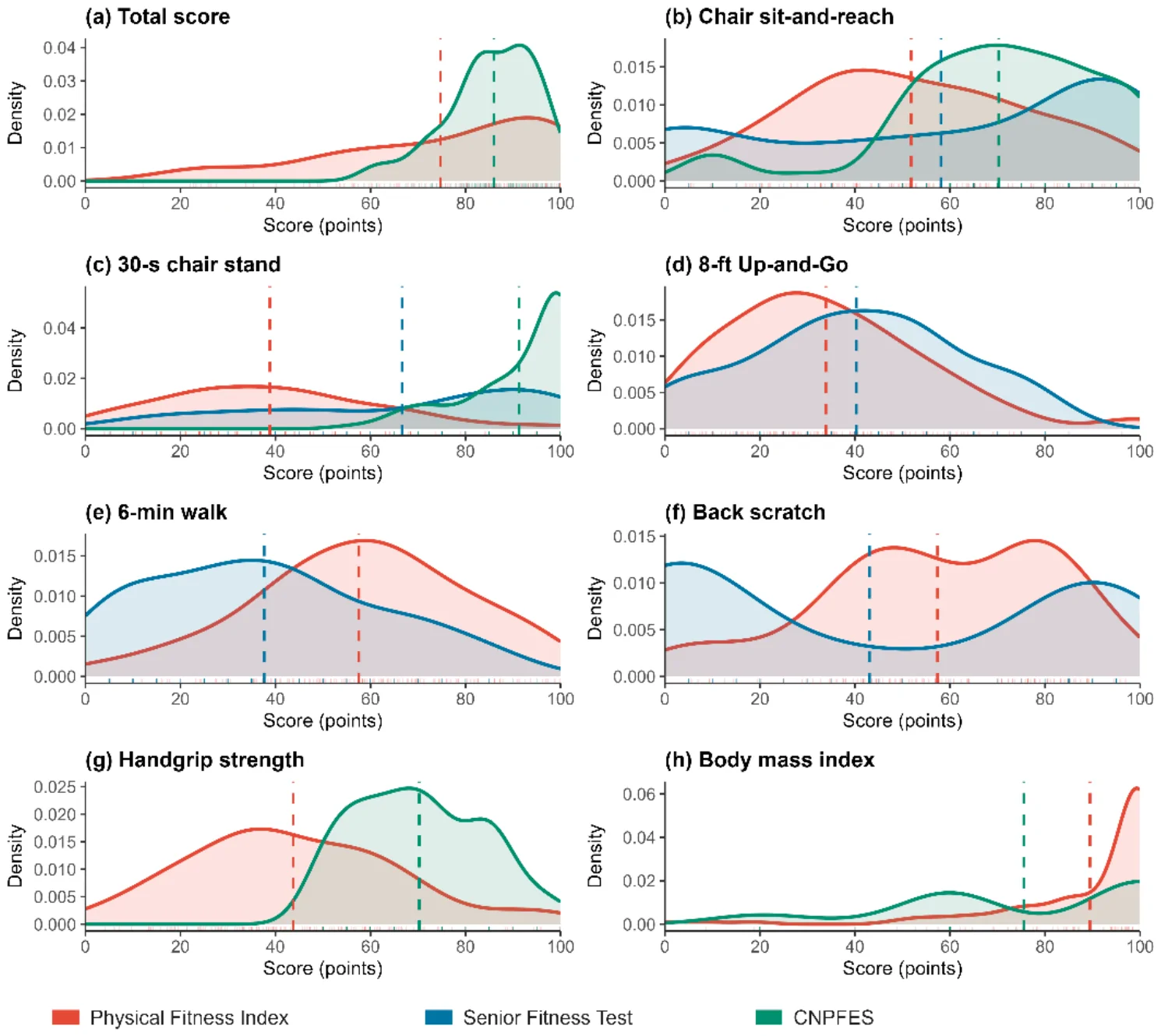
Score distributions for the Physical Fitness Index (red), Senior Fitness Test (blue), and China National Physical Fitness Evaluation Standards (green): (**a**) total score; (**b**) chair sit-and-reach; (**c**) 30 s chair stand; (**d**) 8-ft Up-and-Go; (**e**) 6 min walk; (**f**) back scratch; (**g**) handgrip strength; and (**h**) body mass index. Dashed lines indicate group means.

Figure 3 compares score distributions among the three systems in participants aged 75–79 years. The corrected PFI total score was more widely distributed than the CNPFES score (74.76 ± 23.78 vs. 85.95 ± 9.62; CV = 0.318 vs. 0.112). Because the systems use different tests, calibration procedures, and scoring rules, their absolute scores are not directly equivalent.

Table 4 summarizes the distributional characteristics. After reverse normalization so that a higher score represented better performance, the PFI score for the 8-ft Up-and-Go was 33.89 ± 20.99 (CV = 0.619), compared with 40.29 ± 22.01 for the SFT (CV = 0.546).

The CNPFES 30 s chair-stand score was negatively skewed (skewness = −1.231) and showed a 70.8% ceiling effect, whereas the corresponding PFI score showed ceiling and floor effects of 2.5% and 8.4%, respectively. For the chair sit-and-reach, variability was greater with the SFT than with the PFI (CV = 0.639 vs. 0.475).

For mobility and endurance, the PFI and SFT CVs were 0.619 and 0.546 for the 8-ft Up-and-Go and 0.395 and 0.649 for the 6 min walk, respectively. The SFT back-scratch score had the greatest dispersion (CV = 0.959) and a 42.0% floor effect. The PFI handgrip score was more variable than the CNPFES score (CV = 0.503 vs. 0.192), whereas the PFI BMI score was less variable (CV = 0.210 vs. 0.363) but had a larger ceiling effect (68.0% vs. 50.5%).

Thus, the scoring systems produced materially different distributions. The PFI often showed greater dispersion, but floor and ceiling effects varied by indicator; these descriptive comparisons do not establish superior validity or diagnostic accuracy.

### 3.4. Applications of the Physical Fitness Index

The PFI retained eight prespecified domains represented by BMI, the 30-Second Chair Stand Test, handgrip strength, the 6-Minute Walk Test, the Berg Balance Scale, the 8-Foot Up-and-Go Test, the Chair Sit-and-Reach Test, and the Back Scratch Test. No post hoc dimensionality-reduction procedure was applied. In the development sample, the corrected PFI showed greater score dispersion than the comparison systems for the total score and several indicators, whereas floor and ceiling effects varied by indicator and scoring system. These descriptive differences do not demonstrate superior validity or discriminatory accuracy.

Because the CNPFES and SFT provide age- and sex-specific scoring tables, a simplified equation was derived to approximate the geometric PFI:

$$\begin{array}{cc} Estimated\;PFI & \\ & =\;-109.66\;+\;0.05\;Walk\;+\;0.31SitReach\;-\;0.02BMI\;\\ & +\;0.99\;Stand\;+\;0.79Berg\;+\;0.58Handgrip\;\\ & +\;0.20\;Scratch+\;0.75Age\;+\;3.85Female.\;R^{2}=0.82 \end{array}$$

The equation estimates the geometric PFI and does not redefine its component scoring. Female was coded as 1 and male as 0. The age coefficient is a calibration term arising from sex- and age-stratified normalization and must not be interpreted as evidence that ageing improves physical fitness.

The reduced model explained 82.5% of PFI variance (R^2^ = 0.825; adjusted R^2^ = 0.822). Ten-fold cross-validation yielded an RMSE of 5.79 points, an MAE of 4.33 points, and an out-of-fold R^2^ of 0.814. VIFs were below 2.21. BMI was retained to represent body composition but was not independently significant (*p* = 0.791; Table 5).

## 4. Discussion

This study developed a multidimensional PFI that integrates body composition, strength, endurance, balance, mobility, and flexibility into a single index. Candidate indicators were selected on the basis of data completeness, safety, ease of administration, score dispersion, and overlap with other candidate tests. After correction of the 8-ft Up-and-Go scoring direction, the PFI continued to show a broad distribution in the development sample. However, floor and ceiling effects varied across indicators, and these distributional characteristics should not be interpreted as evidence of superior validity.

Unlike systems that report separate test scores or categorical grades, the PFI provides a continuous total score alongside a domain profile. Given that functional-fitness reference values vary substantially across populations [24], the PFI is best regarded as a complement to, rather than a replacement for, established batteries.

Interpreted within a broader framework of physical function, the PFI’s eight components are conceptually consistent with dimensions of frailty and intrinsic capacity that have been linked to disability, falls, and loss of independence in older adults [4,5,6,25]. Reduced strength, endurance, and balance rarely occur in isolation; contemporary models of healthy ageing treat these domains as interrelated markers of an individual’s overall functional reserve rather than as isolated deficits [6,25]. Viewed from this perspective, pairing a single summary score with a domain-specific profile is intended to capture this interrelatedness in a way that individual field tests, taken alone, cannot.

The PFI generally produced more dispersed scores than the SFT and CNPFES in this sample, although floor and ceiling effects varied by indicator and scoring system. Such differences most likely reflect the distinct tests, normalization procedures, and scoring rules used by the three systems and do not demonstrate greater validity or diagnostic accuracy.

Once validated, the domain profile could help identify which functional deficits—for example, reduced lower-limb power, poor balance, or limited upper-body flexibility—are driving a lower overall score, thereby supporting individually tailored exercise prescription; multicomponent exercise and nutritional interventions have already been shown to improve physical function in prefrail older adults [26]. The total score, in turn, could provide a concise summary for community-level screening and surveillance. Because individual component measures such as handgrip strength have independently been linked to all-cause and cardiovascular mortality in community-dwelling older adults [27,28], and because trajectories of intrinsic capacity are associated with the subsequent onset of disability in activities of daily living [29,30,31], a validated PFI could eventually complement these single-indicator approaches by summarizing risk across domains simultaneously rather than one test at a time. For now, however, repeated use of the PFI to monitor change remains provisional: test–retest reliability, measurement error, and responsiveness to clinically meaningful change have not yet been established, and all three depend on standardizing the testing protocol across repeated administrations [32,33,34].

The differences observed relative to Portuguese [35] and Brazilian [36] samples of older women are consistent with a broader literature reporting substantial cross-national variation in functional-fitness reference values, including comparisons involving very old Japanese cohorts and systematic reviews of benchmark values [37,38]. Such variation likely reflects habitual physical activity and sedentary behaviour, occupational and domestic activity, access to exercise facilities, socioeconomic conditions, and cultural attitudes toward ageing and exercise [8,9,10,15], together with differences in age distribution, sex composition, education, comorbidity, survival characteristics, recruitment methods, and test administration between studies [39]. Reference values and calibration limits derived in one population should therefore not be transferred directly to another without local validation.

Sample-based min–max normalization made it possible to combine indicators expressed in different units, but it also tied the PFI to the observed ranges of each sex-by-age subgroup. The current PFI should therefore be regarded as an internally calibrated relative score rather than a population norm; larger, more representative samples will be needed to establish stable external calibration, ideally reported according to established standards for evaluating and describing the measurement properties of health-status instruments [24,40].

The positive age coefficient in the surrogate equation should not be read as evidence that physical fitness improves with age. Because each component was normalized within sex-specific 5-year age groups, the PFI reflects performance relative to same-age peers rather than absolute performance. An older participant may thus obtain a higher normalized score than a younger participant with identical raw performance, simply because the older subgroup has lower reference bounds. Age therefore functions as a calibration term when raw measurements are used to approximate the age-standardized PFI, not as a marker of improving fitness.

Several limitations should be acknowledged. Participants were recruited by convenience from a single retirement community and represented a relatively healthy, ambulatory subgroup of older adults; the data were cross-sectional, MMSE scores were incomplete, and test order was not fixed. Formal psychometric and external validation were not undertaken, and the study could not assess change over time or predict disability, hospitalization, or mortality—outcomes for which intrinsic-capacity- and function-based measures have shown predictive value in other cohorts [29,30,31,33,34,37,41]. The radar-area score, like other polygon-based multivariate displays, may also vary with axis order and can conceal different component profiles [23]; therefore, the total score should be interpreted with its domain profile.

Overall, the PFI shows promise as a complement to existing systems, pairing an overall score with domain-specific information; multicenter longitudinal studies are now needed before it can be used routinely in clinical or population-level settings.

## 5. Conclusions

In relatively healthy, community-dwelling older adults, this study developed a multidimensional Physical Fitness Index (PFI) that condenses eight domains of physical fitness into both an overall score and a domain-specific profile. By retaining information on specific functional strengths and limitations alongside a continuous summary score, the PFI offers a potential complement to existing assessment systems.

The present findings represent initial index development and preliminary evaluation within the development sample. Because the PFI uses sample-dependent normalization and has not undergone external or longitudinal validation, its application to other populations and its use for monitoring change remain provisional. Multicenter longitudinal studies are required to establish reliability, validity, responsiveness, stable calibration limits, and clinically meaningful thresholds before routine clinical or population-level use.

## Figures and Tables

**Table 1:** Subject information.

	Female	Male	Total	*p*
N	346	215	561	
Age (year)	79.64 ± 5.96	81.93 ± 4.97	80.52 ± 5.71	<0.001
Handgrip strength (kg)	19.85 ± 4.03	27.97 ± 6.25	22.96 ± 6.37	<0.001
BMI (kg/m^2^)	23.54 ± 3.17	24.57 ± 3.32	23.94 ± 3.27	<0.001
Back scratch (cm)	−3.91 ± 10.16	−14.08 ± 15.26	−7.81 ± 13.31	<0.001
Chair sit-and-reach (cm)	3.28 ± 9.89	−5.80 ± 12.88	−0.20 ± 11.97	<0.001
8 ft up-and-go (s)	7.26 ± 1.86	7.96 ± 2.72	7.53 ± 2.26	<0.001
6 min walk test (m)	405.84 ± 88.37	396.54 ± 95.66	402.28 ± 91.26	0.249
30-Second Chair Stand Test (no.)	14.67 ± 4.55	14.02 ± 5.13	14.42 ± 4.78	0.128
Arm curl (no.)	16.36 ± 4.32	15.99 ± 5.43	16.22 ± 4.77	0.406
Berg Balance Test (score)	53.12 ± 3.12	52.60 ± 3.12	52.93 ± 3.13	0.058
2 min Step Test (no.)	82.25 ± 23.28	79.31 ± 22.27	81.12 ± 23.17	0.176
MMSE score (points)	27.70 ± 3.17 (n = 289)	27.63 ± 3.54 (n = 169)	27.68 ± 3.30 (n = 458)	0.834

**Table 2:** Sport test indicators.

Indicators	Sport Test
Body composition	BMI
Lower body strength	30-Second Chair Stand Test
Upper body strength	Arm curl, Handgrip strength
Endurance	6 min walk test, 2 min Step Test
Static balance	Berg Balance Test
Dynamic balance	8-ft up-and-go walk
Lower body flexibility	Chair sit-and-reach
Upper body flexibility	Back scratch

**Table 3:** Descriptive characteristics and within-domain correlations of candidate indicators.

Functional Domain	Candidate Indicator	Valid n	Missing n	CV (%)	IQR	Spearman ρ (*p* Value)
Aerobic endurance	6-Minute Walk Test	561	0	22.69	118.0 m	0.554 (<0.001)
Aerobic endurance	2-Minute Step Test	469	92	28.56	32.0 steps	0.554 (<0.001)
Upper-body strength	Handgrip strength	561	0	27.74	8.0 kg	0.278 (<0.001)
Upper-body strength	Arm Curl Test	554	7	29.43	6.0 repetitions	0.278 (<0.001)

Note: CV, coefficient of variation; IQR, interquartile range. Both statistics describe score dispersion.

**Table 4:** Distributional characteristics of functional fitness indicators across the Physical Fitness Index, Senior Fitness Test, and China National Physical Fitness Evaluation Standards.

Indicator	Method	n	Mean	sd	IQR	CV	Skewness	Kurtosis	Ceiling	Floor
Total Score	China National Physical Fitness Evaluation Standards	118	85.952	9.616	12.547	0.112	−0.657	−0.015	0.381	0.000
	Physical fitness index	99	74.760	23.780	37.483	0.318	−0.793	−0.398	0.333	0.000
30s Chair Stand	China National Physical Fitness Evaluation Standards	120	91.292	11.292	15.000	0.124	−1.231	0.496	0.708	0.000
	Senior Fitness Test	120	66.667	29.243	50.000	0.439	−0.537	−1.069	0.350	0.050
	Physical fitness index	119	38.824	22.398	28.632	0.577	0.454	−0.167	0.025	0.084
Chair Sit-and-Reach	China National Physical Fitness Evaluation Standards	121	70.248	22.331	25.000	0.318	−0.909	0.839	0.231	0.058
	Senior Fitness Test	121	58.099	37.144	70.000	0.639	−0.458	−1.388	0.388	0.215
	Physical fitness index	119	51.822	24.606	33.381	0.475	0.067	−0.764	0.067	0.050
8-ft Up-and-Go	Senior Fitness Test	121	40.289	22.007	30.000	0.546	−0.052	−0.790	0.000	0.132
	Physical fitness index	115	33.892	20.987	27.895	0.619	0.781	0.667	0.026	0.130
6-Minute Walk	Senior Fitness Test	120	37.625	24.400	40.000	0.649	0.323	−0.801	0.017	0.192
	Physical fitness index	118	57.571	22.734	30.428	0.395	−0.267	−0.326	0.085	0.025
Back Scratch	Senior Fitness Test	119	43.067	41.288	90.000	0.959	0.176	−1.804	0.269	0.420
	Physical fitness index	119	57.356	24.963	36.111	0.435	−0.460	−0.518	0.050	0.067
Handgrip Strength	China National Physical Fitness Evaluation Standards	121	70.248	13.492	25.000	0.192	0.251	−0.905	0.091	0.000
	Physical fitness index	119	43.747	22.009	30.711	0.503	0.387	−0.172	0.034	0.050
Body Mass Index	China National Physical Fitness Evaluation Standards	103	75.534	27.430	40.000	0.363	−0.637	−0.794	0.505	0.000
	Physical fitness index	103	89.474	18.787	14.046	0.210	−2.660	8.038	0.680	0.019

CV reflects relative score dispersion. Skewness and kurtosis describe distributional shape. Ceiling and floor effects are the proportions scoring ≥90 and ≤10 points, respectively; values >0.15 were considered notable.

**Table 5:** Reduced regression model for estimating the Physical Fitness Index.

Term	Estimate	SE	95% CI Low	95% CI High	t	*p*	VIF
Intercept	−109.6352	7.0982	−123.5780	−95.6923	−15.45	<0.001	—
6-Minute Walk	0.0456	0.0036	0.0386	0.0526	12.80	<0.001	1.84
Chair Sit-and-Reach	0.3080	0.0234	0.2621	0.3539	13.18	<0.001	1.37
Body Mass Index	−0.0215	0.0809	−0.1805	0.1375	−0.27	0.791	1.22
30-Second Chair Stand	0.9945	0.0627	0.8713	1.1177	15.86	<0.001	1.57
Berg Balance Scale	0.7931	0.0938	0.6089	0.9773	8.46	<0.001	1.50
Handgrip Strength	0.5807	0.0552	0.4723	0.6891	10.52	<0.001	2.16
Back Scratch	0.2043	0.0214	0.1623	0.2463	9.56	<0.001	1.41
Age	0.7536	0.0489	0.6576	0.8496	15.42	<0.001	1.36
Female	3.8478	0.7312	2.4115	5.2840	5.26	<0.001	2.21

## Data Availability

The original contributions presented in the study are included in the article, further inquiries can be directed to the corresponding author/s.

## References

[1] Zhao Y., Chen P., Gong Y., Gu Z., Zhou W. (2024). Functional fitness and physical independence in later years: Cut-off values and validation. BMC Geriatr..

[2] National Bureau of Statistics of China (2026). Statistical Communiqué of the People’s Republic of China on the 2025 National Economic and Social Development.

[3] United Nations Department of Economic and Social Affairs, Population Division (2024). World Population Prospects 2024: Summary of Results.

[4] Yang X., Li S., Xu L., Liu H., Li Y., Song X., Bao J., Liao S., Xi Y., Guo G. (2024). Effects of multicomponent exercise on frailty status and physical function in frail older adults: A meta-analysis and systematic review. Exp. Gerontol..

[5] Corral-Pérez J., Mier A., Vázquez-Sánchez M.Á., Naranjo-Márquez M., Ponce-Gonzalez J.G., Casals C. (2024). Multidimensional associations of physical performance, balance, wellness and daily activities with frailty in older adults with coexisting frailty and diabetes. J. Clin. Nurs..

[6] Beard J.R., Officer A., de Carvalho I.A., Sadana R., Pot A.M., Michel J.-P., Lloyd-Sherlock P., Epping-Jordan J.E., Peeters G.M.E.E.G., Mahanani W.R. (2016). The World report on ageing and health: A policy framework for healthy ageing. Lancet.

[7] Yang H., Li J., Shan W., Ren S., Chai X., Lu J., Yan H., Lu Y. (2025). The effect of different exercise modalities on older adults’ quality of life: An assessor-blinded randomized controlled trial. Sci. Rep..

[8] Parra-Rizo M.A., Díaz-Toro F., Hadrya F., Pavón-León P., Cigarroa I. (2022). Association of co-living and age on the type of sports practiced by older people. Sports.

[9] Agustí A.I., Guillem-Saiz J., González-Moreno J., Cantero-García M., Cigarroa I., Parra-Rizo M.A. (2023). Predictors of health satisfaction in Spanish physically active older adults: A cross-sectional observational study. Geriatrics.

[10] World Health Organization (2022). Global Status Report on Physical Activity 2022.

[11] Osness W.H., Adrian M., Clark B., Hoeger W.W., Raab D., Wiswel I.R. (1990). Functional Fitness Assessment for Adults Over 60 Years: A Field-Based Assessment.

[12] Rikli R.E., Jones C.J. (1999). Development and validation of a functional fitness test for community-residing older adults. J. Aging Phys. Act..

[13] China National Physical Fitness Monitoring Center (2023). China National Physical Fitness Evaluation Standards (2023 Revision).

[14] Guralnik J.M., Simonsick E.M., Ferrucci L., Glynn R.J., Berkman L.F., Blazer D.G., Scherr P.A., Wallace R.B. (1994). A short physical performance battery assessing lower extremity function: Association with self-reported disability and prediction of mortality and nursing home admission. J. Gerontol..

[15] Santos D.A., Silva A.M., Baptista F., Santos R., Vale S., Mota J., Sardinha L.B. (2012). Sedentary behavior and physical activity are independently related to functional fitness in older adults. Exp. Gerontol..

[16] World Health Organization (2017). Integrated Care for Older People: Guidelines on Community-Level Interventions to Manage Declines in Intrinsic Capacity.

[17] Thompson P.D., Arena R., Riebe D., Pescatello L.S. (2013). ACSM’s new preparticipation health screening recommendations from ACSM’s guidelines for exercise testing and prescription, ninth edition. Optom. Vis. Sci..

[18] Folstein M.F., Folstein S.E., McHugh P.R. (1975). “Mini-mental state”: A practical method for grading the cognitive state of patients for the clinician. J. Psychiatr. Res..

[19] Rikli R.E., Jones C.J. (2013). Senior Fitness Test Manual.

[20] ATS Committee on Proficiency Standards for Clinical Pulmonary Function Laboratories (2002). ATS statement: Guidelines for the six-minute walk test. Am. J. Respir. Crit. Care Med..

[21] Berg K. (1989). Measuring balance in the elderly: Preliminary development of an instrument. Physiother. Can..

[22] Dunsky A., Ayalon M., Netz Y. (2011). Arm-curl field test for older women: Is it a measure of arm strength?. J. Strength Cond. Res..

[23] Saary M.J. (2008). Radar plots: A useful way for presenting multivariate health care data. J. Clin. Epidemiol..

[24] Mokkink L.B., Terwee C.B., Patrick D.L., Alonso J., Stratford P.W., Knol D.L., Bouter L.M., de Vet H.C.W. (2010). The COSMIN checklist for assessing the methodological quality of studies on measurement properties of health status measurement instruments: An international Delphi study. Qual. Life Res..

[25] Cesari M., De Carvalho I.A., Thiyagarajan J.A., Cooper C., Martin F.C., Reginster J.-Y., Vellas B., Beard J.R. (2018). Evidence for the domains supporting the construct of intrinsic capacity. J. Gerontol. Ser. A Boil. Sci. Med. Sci..

[26] Liu C., Xu H., Chen L., Zhu M. (2022). Exercise and nutritional intervention for physical function of the prefrail: A systematic review and meta-analysis. J. Am. Med. Dir. Assoc..

[27] Hsu N.-W., Lin C.-H., Yang N.-P., Chen H.-C., Chou P. (2023). Handgrip strength is associated with mortality in community-dwelling older adults: The Yilan cohort study, Taiwan. BMC Public Health.

[28] López-Bueno R., Andersen L.L., Koyanagi A., Núñez-Cortés R., Calatayud J., Casaña J., Cruz B.d.P. (2022). Thresholds of handgrip strength for all-cause, cancer, and cardiovascular mortality: A systematic review with dose-response meta-analysis. Ageing Res. Rev..

[29] Campbell C.L., Cadar D., McMunn A., Zaninotto P. (2023). Operationalization of intrinsic capacity in older people and its association with subsequent disability, hospital admission and mortality: Results from the English Longitudinal Study of Ageing. J. Gerontol. Ser. A.

[30] Prince M.J., Acosta D., Guerra M., Huang Y., Jacob K.S., Jimenez-Velazquez I.Z., Jotheeswaran A.T., Rodriguez J.J.L., Salas A., Sosa A.L. (2021). Intrinsic capacity and its associations with incident dependence and mortality in 10/66 Dementia Research Group studies in Latin America, India, and China: A population-based cohort study. PLoS Med..

[31] Zhang S., Wu S., Guo R., Ding S., Wu Y. (2024). Patterns of intrinsic capacity trajectory and onset of activities of daily living disability among community-dwelling older adults. J. Glob. Health.

[32] Hopkins W.G. (2000). Measures of reliability in sports medicine and science. Sports Med..

[33] Husted J.A., Cook R.J., Farewell V.T., Gladman D.D. (2000). Methods for assessing responsiveness: A critical review and recommendations. J. Clin. Epidemiol..

[34] Hao Q., Kuspinar A., Griffith L., D’aMore C., Mayhew A.J., Wolfson C., Guyatt G., Raina P., Beauchamp M. (2023). Measuring physical performance in later life: Reliability of protocol variations for common performance-based mobility tests. Aging Clin. Exp. Res..

[35] Marques E.A., Baptista F., Santos R., Vale S., Santos D.A., Silva A.M., Mota J., Sardinha L.B. (2014). Normative functional fitness standards and trends of Portuguese older adults: Cross-cultural comparisons. J. Aging Phys. Act..

[36] Krause M.P., Januário R.S., Hallage T., Haile L., Miculis C.P., Gama M.P., Goss F.L., da Silva S.G. (2009). A comparison of functional fitness of older Brazilian and American women. J. Aging Phys. Act..

[37] Cossio-Bolaños M., Vidal-Espinoza R., Villar-Cifuentes I., de Campos L.F.C.C., de Lázari M.S.R., Urra-Albornoz C., Sulla-Torres J., Gomez-Campos R. (2024). Functional fitness benchmark values for older adults: A systematic review. Front. Public Health.

[38] Matsumoto K., Gondo Y., Masui Y., Yasumoto S., Yoshida Y., Ikebe K., Arai Y., Kabayama M., Kamide K., Akasaka H. (2022). Physical performance reference values for Japanese oldest old: A SONIC study. BMC Geriatr..

[39] Bauman A.E., Reis R.S., Sallis J.F., Wells J.C., Loos R.J.F., Martin B.W. (2012). Lancet Physical Activity Series Working Group. Correlates of physical activity: Why are some people physically active and others not?. Lancet.

[40] Elsman E.B.M., Mokkink L.B., Terwee C.B., Beaton D., Gagnier J.J., Tricco A.C., Baba A., Butcher N.J., Smith M., Hofstetter C. (2024). Guideline for reporting systematic reviews of outcome measurement instruments (OMIs): PRISMA-COSMIN for OMIs 2024. Qual. Life Res..

[41] Navarrete-Villanueva D., Gómez-Cabello A., Marín-Puyalto J., Moreno L.A., Vicente-Rodríguez G., Casajús J.A. (2021). Frailty and physical fitness in elderly people: A systematic review and meta-analysis. Sports Med..

